# Supplementary material for: Gut Microbiome Composition and Variance Are Modified by Degree of Growth Failure in Preterm Infants: A Prospective Study
Source: Nutrients. 2025 Dec 13;17(24):3907. doi: 10.3390/nu17243907 (PMC12735543; doi:10.3390/nu17243907)
Supplement: Supplementary file 1 [file nutrients-17-03907-s001.zip › nutrients-4010918-supplementary.pdf]

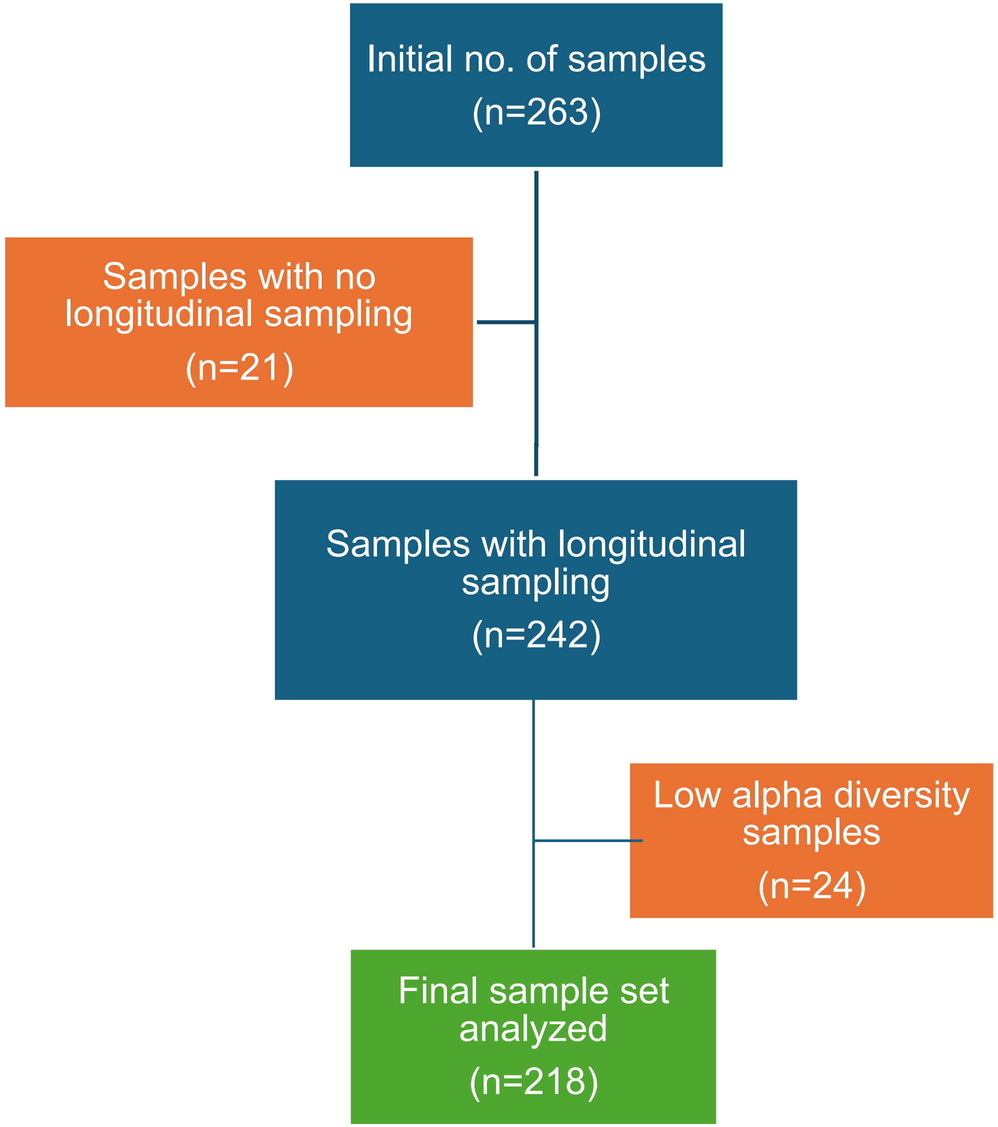

Supplementary Figure S1: Sample Screening Flowchart

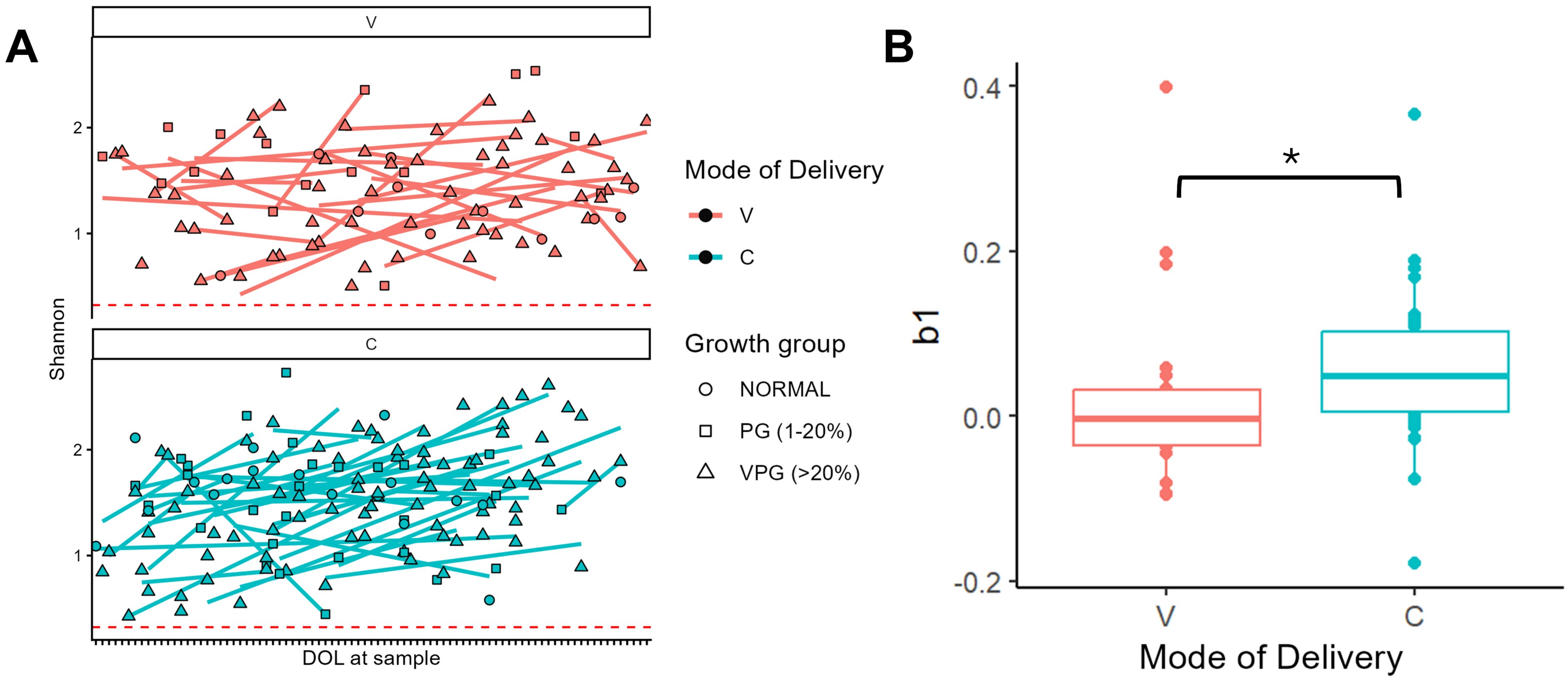
A
V
C
2
1
Shannon
DOL at sample
Mode of Delivery
V
C
Growth group
NORMAL
PG (1-20%)
VPG (>20%)
B
0.4
0.2
0.0
-0.2
b1
*
V
C
Mode of Delivery

Supplementary Figure S2 Legend:

Figure S2: Comparing the slopes of within-individual lines of best fit for infants in the vaginal birth (V) and cesarean section (C) groups. A) shows individual lines of best fit in each birth modality group across days of life post-birth (DOL). Shapes of datapoints correspond to growth percentile bracket of the infant at the time of sample acquisition. B) shows the distribution of slopes (B1) between delivery mode (vaginal [V] and cesarean section [C]) groups, with a significant increase ($p<0.05$) in median slope for infants born by cesarean section.

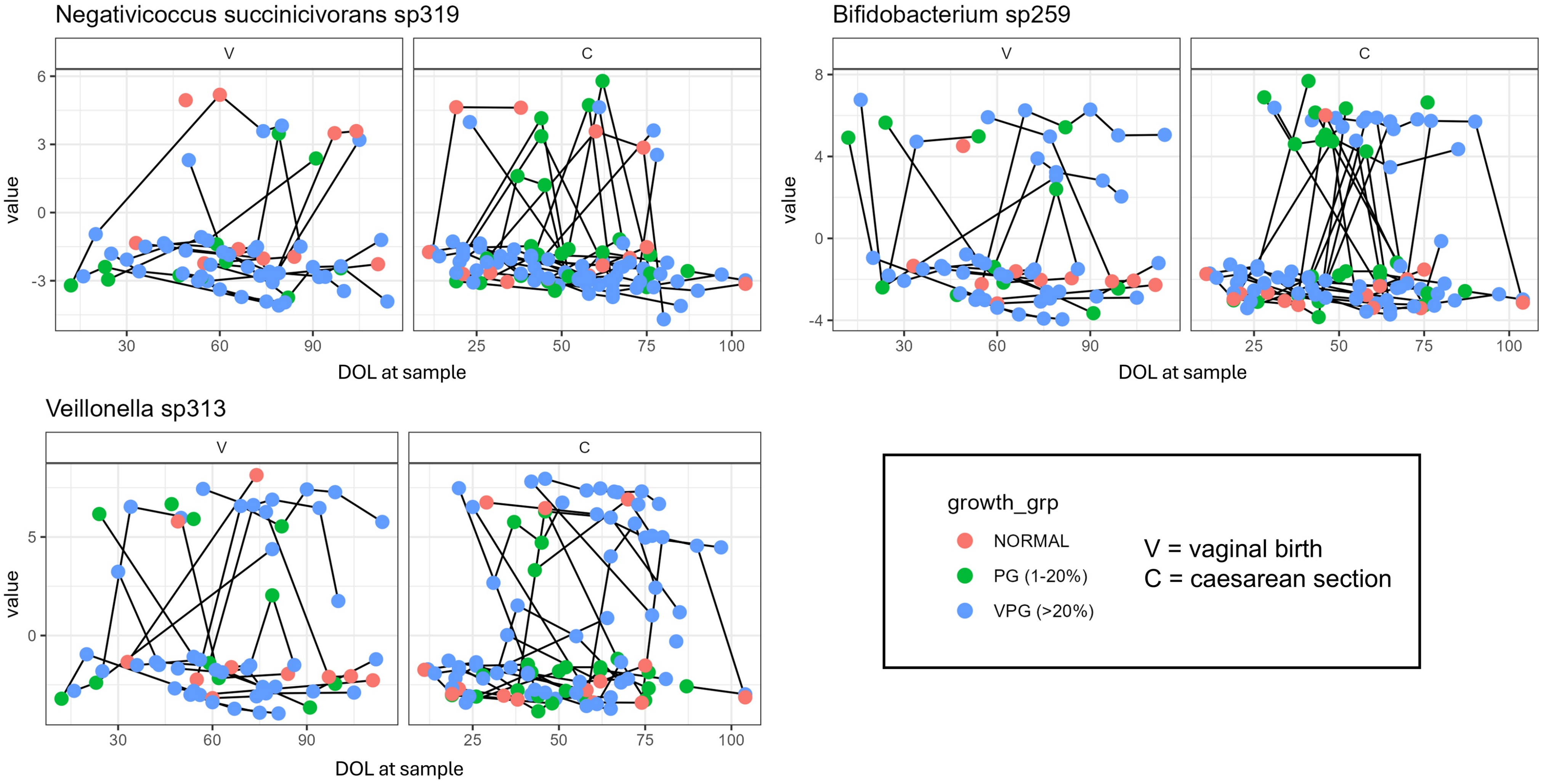
Negativicoccus succinicivorans sp319
V
C
value
6
3
0
-3
30
60
90
25
50
75
100
DOL at sample
Bifidobacterium sp259
V
C
value
8
4
0
-4
30
60
90
25
50
75
100
DOL at sample
Veillonella sp313
V
C
value
5
0
30
60
90
25
50
75
100
DOL at sample
growth_grp
NORMAL
PG (1-20%)
VPG (>20%)
V = vaginal birth
C = caesarean section

Supplementary Figure S3 Legend:

Figure S3: *W*ithin individual variability in the abundance of differentially abundant taxa across early life of preterm infants partitioned by vaginal birth (V) and cesarean section (C) groups. Individual dots are colored by the growth percentile bracket of the infant at time of sample, and each black line represents the trend in ASV abundance for a given infant over time.
